# An experience for road safety applied research training: Tabriz-Iran 

**Published:** 2019-08

**Authors:** Abbas Ali Dorosti, Homayoun Sadeghi-Bazargani, Majid Karamouz, Ahmad Mardi, Mostafa Farahbakhsh, Mahin Dahim, Mohammad Saadati, Najibeh Khoshmaram, Robabeh Emrouzi

**Affiliations:** ^*a*^Anesthesiology Department, Faculty of Medicine, Tabriz university of medical sciences, Tabriz, Iran.; ^*b*^Road Traffic Injury Research Center, Tabriz University of Medical Sciences, Tabriz, Iran.; ^*c*^Provincial Health Center, Tabriz University of Medical Sciences, Tabriz, Iran.

## Abstract

**Background:**

Traffic accidents and their related injuries are important challenges in public health. Driving crashes is determined 8th cause of mortality worldwide trends study of crashes mortality shows that in 2030 traffic accidents will be 5th of mortality causes.

**Methods:**

This study is aimed at empowering and involving juvenile health experts in youth accident prevention. Improving traffic accident prevention skills will be done through an interdisciplinary approach. Theoretical and practical Empowerment workshops were held in collaboration with health deputy of Tabriz University of Medical Sciences for 40 persons. The workshops were attended by experts from the district Health Network and the Sport and Youth Organization of each city.

**Results:**

After the first workshop, four working teams were selected. A research method workshop was organized for this group. A traffic safety research project was defined for each team. These projects were implemented in Tabriz, Jolfa, Azarshahr and Sahand cities.

**Conclusions:**

It seems that project based health system research workshops are effective than traditional research workshops.

**Keywords:**

Health system research, HSR, Traffic safety, Tabriz

